# Enhanced Metakaolin Reactivity in Blended Cement with Additional Calcium Hydroxide

**DOI:** 10.3390/ma15010367

**Published:** 2022-01-04

**Authors:** Kira Weise, Neven Ukrainczyk, Aaron Duncan, Eduardus Koenders

**Affiliations:** Institute of Construction and Building Materials, Technical University of Darmstadt, 64287 Darmstadt, Germany; aaron.duncan@web.de (A.D.); koenders@wib.tu-darmstadt.de (E.K.)

**Keywords:** supplementary cementitious materials (SCMs), pozzolanic reaction, calcined clay, metakaolin, thermogravimetric analysis (TGA), calcium hydroxide

## Abstract

This study aims to increase the pozzolanic reactivity of metakaolin (MK) in Portland cement (PC) blends by adding additional calcium hydroxide (CH_add) to the initial mixture. Cement paste samples were prepared with PC, MK and water with a water-to-binder ratio of 0.6. Cement replacement ratios were chosen from 5 to 40 wt.% MK. For higher replacement ratios, i.e., 20, 30 and 40 wt.% MK, CH_add was included in the mixture. CH_add-to-MK ratios of 0.1, 0.25 and 0.5 were investigated. Thermogravimetric analysis (TGA) was carried out to study the pozzolanic reactivity after 1, 7, 28 and 56 days of hydration. A modified mass balance approach was used to normalize thermogravimetric data and to calculate the calcium hydroxide (CH) consumption of samples with CH_add. Results showed that, without CH_add, a replacement ratio of 30 wt.% or higher results in the complete consumption of CH after 28 days at the latest. In these samples, the pozzolanic reaction of MK turned out to be restricted by the amount of CH available from the cement hydration. The increased amount of CH in the samples with CH_add resulted in an enhanced pozzolanic reaction of MK as confirmed by CH consumption measurements from TGA.

## 1. Introduction

Concrete, with cement as binder, is the most used construction material in the world [1]. However, with a global cement production of around four billion tons per year [2], it is responsible for about 8% of the worldwide androgenic CO_2_ emissions [3]. Because of this, enhanced replacements of Portland cement (PC) by more environmentally friendly alternatives are moving into the focus of the construction industry [4]. Most of these so-called supplementary cementitious materials (SCMs) are fly ash, silica fume and ground granulated blast-furnace slag. However, the change to renewable energy causes the global amount of fly ash [5] and ground granulated blast-furnace slag to significantly decline [6], which underlines the importance for research on SCMs that are available for replacement of PC in large amounts.

In recent years, calcined clay has turned out to be a serious alternative for a pozzolanic SCM that can be used in mortars and concrete [7,8]. Due to the specific morphological characteristics of some particular clays, such as kaolinite, its calcination at relatively low temperatures disclosed great potential to act as a highly reactive component in cementitious systems [7,8,9]. Clays benefit from a vast global availability [10], require relatively low temperatures for calcination (~750 °C) [7,11,12], and emit less CO_2_ [13,14]. The main challenge concerning the use of local calcined clays as SCMs is to control the variation of their composition and its impact on the reactivity [7,9,11,12,15,16,17,18].

Regarding the mineral composition of clay-based materials, various studies have shown that in particular the amount of metakaolin in calcined clays is mainly responsible for their pozzolanic reactivity in cementitious binders [7,12,16]. To better comprehend the complex mineral system of calcined clays, in particular the influence of the varying composition on the pozzolanic performance, it is crucial to investigate the reactive behavior of each individual component. Therefore, this study focuses on the reactivity of metakaolin (MK) in a cement paste system, with emphasis on the amount of calcium hydroxide (CH) needed for the pozzolanic reaction.

Metakaolin is obtained from the dehydroxylation of kaolinite, which occurs during a thermal activation between 400 °C and 800 °C [11,16]. The release of OH^−^ ions results in an amorphous structure, called metakaolin, that is required for a pozzolanic reaction in cementitious systems [15]. The pozzolanic reaction of MK leads to the additional formation of hydrate phases along with the consumption of CH, resulting in an increase in the material strength [19]. Depending on the amount of CH available, the following hydrate phases may be formed [20]:

AS_2_ + 3CH + 6H → C-S-H + C_2_ASH_8_ (1)

AS_2_ + 5CH + 3H → 2C-S-H + C_3_AH_6_ (2)

AS_2_ + 6CH + 9H → 2C-S-H + C_4_AH_13_ (3)

where:

- AS_2_ is metakaolin (Al_2_O_3_ · 2SiO_2_ · 2H_2_O);
- CH is calcium hydroxide (Ca(OH)_2_);
- H is water (H_2_O);
- C-S-H is the collective term for calcium silicate hydrates having wide range of C/S molar ratios (C: CaO; S: SiO_2_);
- C_2_ASH_8_ is hydrated stratlingite;
- C_3_AH_6_ is tricalcium aluminate hydrate;
- C_4_AH_13_ is tetracalcium aluminate hydrate.

The addition of metakaolin in cementitious systems typically results in an increased strength of pastes [21], mortars [21,22,23] and concretes [24,25,26,27], already after a curing period of 28 days. Moreover, the reduction of CH in blended cement systems due to the pozzolanic reaction may implicitly enhance the durability of cementitious systems, e.g., the sulfate resistance [19,28]. Another positive effect of incorporating MK is the reduced total capillary porosity that leads to a lower rate of water absorption of metakaolin-modified mortars [22,24] as well as to a delay in the advancing carbonation front [24]. A lower capillary porosity also results in an increased resistance against chloride penetration [26]. Finally, the consumption of CH by a pozzolanic reaction with MK also prevents the formation of Friedel’s salt [23].

Regarding the compressive strength of metakaolin-modified cementitious systems, optimum cement replacement ratios of 10 wt.% (w/b 0.35) [26], 12.5 wt.% (w/b 0.4) [26] and 20 wt.% (w/b 0.4) [27] were reported for concrete and 10 wt.% (w/b 0.55) [21] and 25 wt.% (w/b 0.6) [23] for mortar. The difference in findings is mainly based on variations in the chemical and mineralogical composition of the used metakaolins [27] that is largely dependent on both the purity and the amount of kaolinite in the raw material [29] as well as on the calcination process [30]. In addition, the type of cement turned out to have a significant influence on the reported test results [28,31]. The relatively low optimum replacement ratios reported, i.e., from 10 to 25 wt.%, should be related to the high pozzolanic activity of MK that reacted with the sufficiently available CH formed by cement hydration. For higher replacement ratios, certain amounts of MK may stay unreacted due to a lack of CH needed for the pozzolanic reaction, consequently causing a lower compressive strength.

Most recently, Briki et al. [28] indicated that the addition of extra calcium hydroxide in a blended binder paste (CH_add/MK~0.36) results in a higher degree of reaction of MK, but only up to seven days of curing, followed by a stagnation at later ages. This experiment was performed to prove the hypothesis that a lack of CH may most likely not be the only reason for the declining MK reaction at later ages, consisting of a limestone–calcined-clay–cement blend (the so-called LC^3^) system. However, different from the LC³-system considered by Briki et al. [32], when focusing on relatively low replacement ratios of PC by MK (~22.3 wt.%), the present paper reports preliminary findings on the reactivity of metakaolin-to-cement binders with high replacement ratios. The hypothesis to be confirmed is that, for high replacement ratios in cement-metakaolin systems, the amount of calcium hydroxide generated by the alite and belite reactions may become decisive in the pozzolanic reaction, as CH is a critical reactant (educt), and thus may also define a lower threshold for the replacement ratio [28]. Therefore, the aim of this study is to investigate the pozzolanic reactivity of MK for high replacement ratios in cement–metakaolin systems (20, 30 and 40 wt.%) by adding extra calcium hydroxide (CH_add) to the initial paste mixture. The proposed approach could possibly clarify the reduction in compressive strength for high cement replacement ratios of MK and show possible improvements for durability, where the additional CH may assure sufficient alkalinity in a reinforced concrete needed for an effective passivation of steel.

## 2. Materials and Methods

### 2.1. Materials

**Figure 1:**
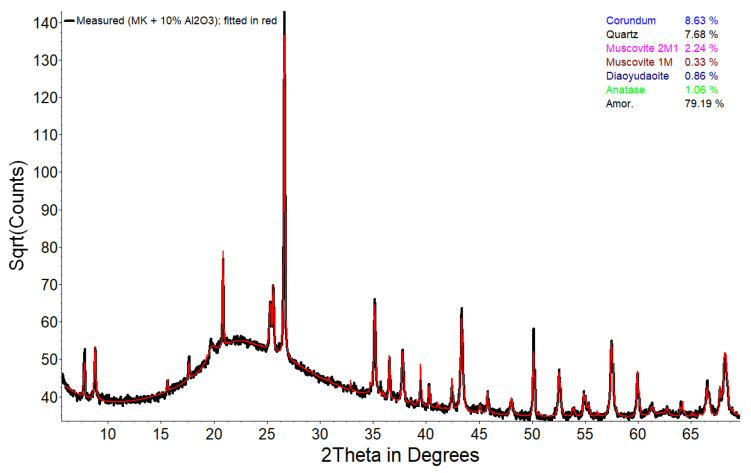
Quantitative phase analysis of the metakaolin sample spiked with corundum (MK + 10% corundum as internal standard with diaoyudaoite as impurity) by means of Rietveld refinement simulation of powder X-ray diffractogram: measurement (black line) and fitted convoluted model calculated from contributions of individual phases (red line).

The metakaolin (MK) employed in this research was an almost pure industrial type of metakaolin that was produced by grinding calcined kaolinite-rich clay originating from secondary geological deposits (source unknown). It was calcinated in an industrial Herreshoff (multiple hearth) furnace with controlled calcination temperature (<750 °C) and time to ensure a homogenous high reactivity product. It has a specific density of 2.6 g/cm³ and a specific surface of 200,000 cm²/g (nitrogen sorption). The Rietveld refinement X-ray diffraction result of a MK sample (spiked with corundum as internal standard) is shown in Figure 1. Phase quantification was obtained using Topas version 5 software from Bruker (Billerica, MA, USA) and resulted in 87.4 wt.% amorphous material (~metakaolin), 8.5 wt.% quartz, 2.9 wt.% muscovite and 1.2 wt.% anatase (in original MK sample). “Sulfo 5 R” produced by Holcim (Deutschland) GmbH (Hamburg, Germany) was chosen as Portland cement (PC) CEM I 52.5 R. The chemical compositions of MK and PC are outlined in Table 1. Powdered calcium hydroxide (CH) (≥96%) with a specific density of 2.24 g/cm³ was obtained from Carl Roth GmbH & Co. KG (Karlsruhe, Germany).

### 2.2. Sample Preparation

**Figure 2:**
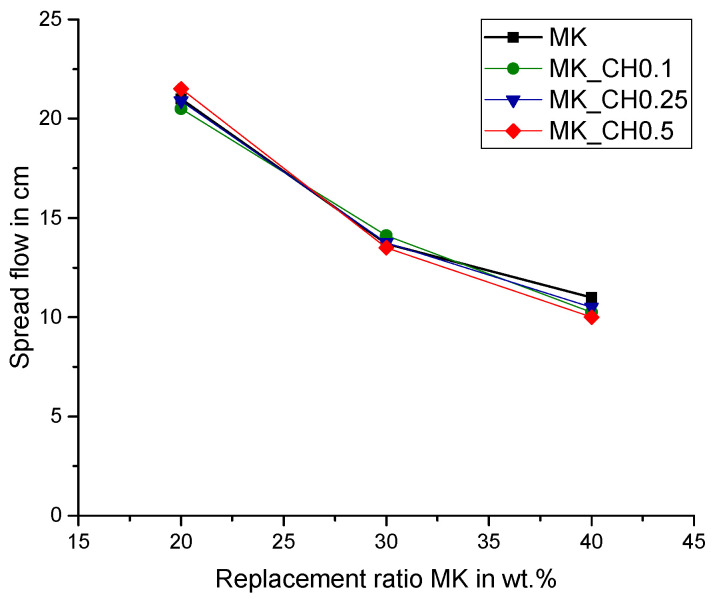
Spread flow of the consistency of the paste samples with high replacement ratios (20, 30 and 40 wt.% MK).

In the experimental program, paste samples were prepared containing Portland cement (PC) and metakaolin (MK) with cement replacement ratios of 5, 10, 20, 30 and 40 wt.% MK. For the higher cement replacement ratios (20, 30 and 40 wt.%) additional calcium hydroxide (CH_add) was added to the initial paste mixture as shown in Table 2. To ensure a good workability of the pastes, even for high replacement ratios and without employing superplasticizers, a water-to-binder ratio (w/b) of 0.6 was applied. In this case, the binder includes PC, MK and CH_add. The consistency of the paste samples was measured according to DIN EN 1015-3, but without jolting the spreading table 15 times. The results for the samples with high replacement ratios (20, 30 and 40 wt.% MK) are shown in Figure 2.

The CH_add-to-MK ratios (CH_add/MK) were chosen as 0.1, 0.25 and 0.5. For comparison, in literature values of 0.36 [32] and 0.37 [28] were used. In those two studies, mixed calcined clays with an amount of 48 wt.% [32] and 45 wt.% metakaolin [28] were investigated in cement paste and mortar systems, along with additionally added calcium hydroxide.

A suspension mixer from BAUER MAT Slurry Handling Systems (Immenstadt, Germany) was used to prepare the cement paste samples. The powder precursors were mixed with tap water (20 ± 1 °C) until a homogeneous paste was achieved. Paste samples were mixed for one minute at 60 Hz, filled into 10 mL sealed polypropylene containers and were stored at 20 °C. To evaluate the reaction process over time, hydration was stopped after 1, 7, 28 and 56 days. For hydration stoppage, the hardened samples were crushed, hand-milled and washed three times with 5 mL of acetone (>99.5%) while continuing milling according to [33].

### 2.3. Measurement Methods

After reaching each hydration time (1, 7, 28 and 56 days), a thermogravimetric analysis (TGA) was performed using a ”STA 449 F5 Jupiter” from Erich NETZSCH GmbH & Co. Holding KG (Selb, Germany). Nitrogen was chosen as inert gas and the crucible consisting of aluminum oxide was filled with 40 to 50 mg of powdered sample. Each sample was first heated from room temperature to 40 °C, kept constant at this temperature for 30 min and then heated up to 1000 °C at a constant heating rate of 20 °C per minute [34].

### 2.4. Mass Balance Approach for Normalization of TGA Data from Samples with CH_add

**Figure 3:**
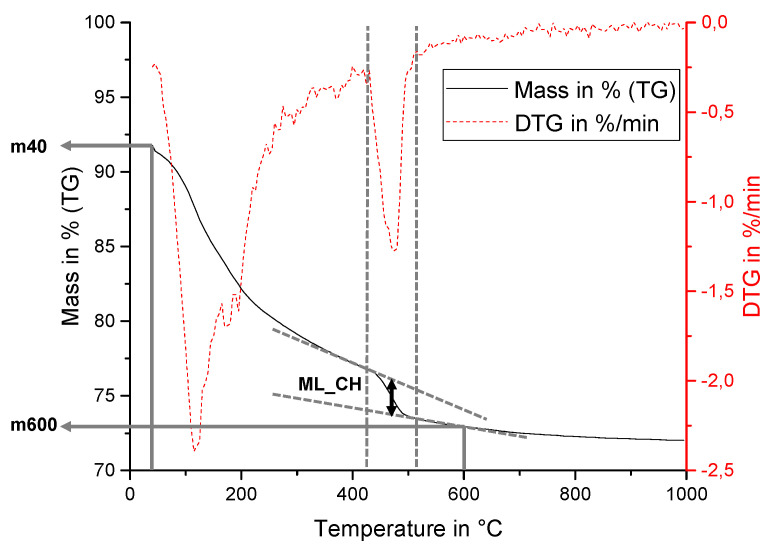
An example of typical TG and DTG curves of metakaolin–cement paste samples (MK10 after 56 days) showing the parameters used for the analysis.

In this section, the evaluation of data is reported, which was achieved from the thermogravimetric analysis (TGA) of the paste samples used from this study. A well-established method was employed to calculate and normalize the amount of CH in the samples of binder systems with cement and metakaolin. The method was slightly adapted for evaluating metakaolin-cement paste samples that contain additionally added CH (CH_add). Figure 3 shows an example for typical TG curve and its derivative (=DTG curve) of the metakaolin–cement paste sample MK10 after 56 days of hydration. The parameters used for the analysis of the paste samples in Equations (4)–(6) and (8) (m40, m600 and ML_CH) are outlined in Figure 3, as well as the tangential approach for ML_CH.

As a first step, raw thermogravimetric data were corrected to the measured mass at 40 °C (m40) according to Equation (4), so that the 100 wt.% sample is related to a temperature of 40 °C [34]:

(4) $$x^{*}=\frac{x}{m40}\;\cdot \;100\;\mathrm{wt}.\%$$

where:

- x^*^ is the corrected TGA value in wt.%;
- x is any TGA data (mass or mass loss) in wt.%;
- m40 is the mass at 40 °C taken from TGA data in wt.% (see Figure 3).

The mass loss at around 400 °C to 500 °C was assigned to the dehydroxylation of calcium hydroxide (which is in line with the literature [9,34,35,36,37]). The temperature range was set for each sample individually by using the derivation of the TG curve (=DTG curve). The tangential method according to Lothenbach et al. [35] was employed to calculate the amount of calcium hydroxide in each sample according to Equation (5), while considering the following molar masses: Ca(OH)_2_: 74 g/mol; H_2_O: 18 g/mol. Samples without CH_add were normalized to the thermogravimetrically measured mass at 600 °C (m600^*^) according to Equation (5) as established in the literature [33,35]:

(5) $$CH=\frac{{\mathrm{ML}\_\mathrm{CH}}^{*}}{{m600}^{*}}\;\cdot \;\frac{74\;g/\mathrm{mol}}{18\;g/\mathrm{mol}}\cdot \;100$$

where:

- *CH* is the amount of calcium hydroxide in the sample (t = x) in g/100 g binder;
- ML_CH^*^ is the corrected mass loss between approximately 400 °C and 500 °C; determined with the tangential method from TGA data in wt.% (ML_CH shown in Figure 3, corrected according to Equation (4));
- m600^*^ is the corrected mass at 600 °C taken from TGA data in wt.% (m600 shown in Figure 3, corrected according to Equation (4)), typically representing the binder mass, in this paper also referred to as the normalization value as it normalizes the *CH* results relative to the binder (i.e., to have units in g/100 g binder). To account for the added CH (CH_add) as part of the binder, this value has to be replaced by a corrected one, m600^*(1)^ or m600^*(2)^ as detailed in the following descriptions and calculated according to Equation (6) or (8).

To normalize the samples that contained additional CH (CH_add), two different scenarios were introduced. The first one assumed that CH_add stays constant over the hydration time and the pozzolanic reaction only consumes the CH produced by cement hydration (CH_PC): CH_add (t = x) = CH_add (t = 0). In the second scenario, the entire amount of CH_PC was assumed to be consumed, so that CH_add decreases with progress of the pozzolanic reaction of MK: CH_add (t = x) < CH_add (t = 0).

**Figure 4:**
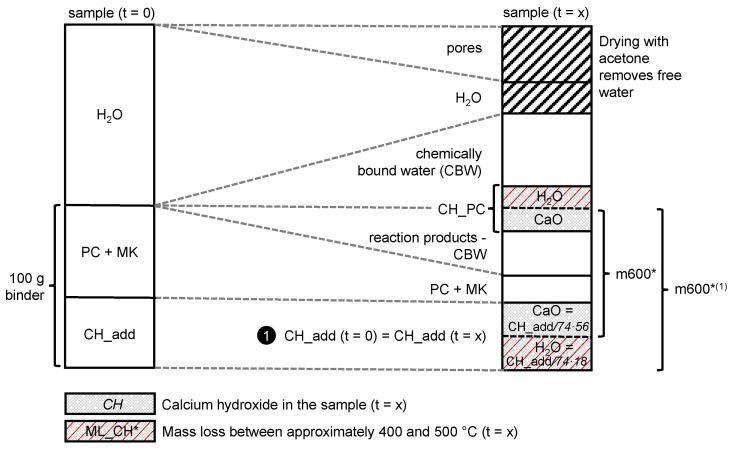
Scheme explaining the calculation of the normalization value m600^*(1)^ for TGA of samples with CH_add according to scenario 1, assuming that CH_add is not consumed by pozzolanic reaction of MK. Note that the linear connection between t = 0 and t = x is only a simplification that does not play a role for the calculation of the normalization value m600^*(1)^. The dimensions are not realistic; they are chosen for a clear visualization only.

The amount of CH_add was taken into account to normalize the TGA data to 100 g binder that contains PC, MK and CH_add. For the analysis of the TGA data from the samples with CH_add, scenario 1 or 2 was selected by checking if the *Consumed CH* (calculation according to Equation (7) described in detail below) was less than the CH produced by cement hydration (CH_PC). To check this, scenario 1 was calculated first, where the normalization value (m600^*(1)^) was adjusted for the stoichiometrically calculated amount of water in CH_add, according to Equation (6). A graphical explanation of how the normalization value for scenario 1 (m600^*(1)^) was calculated is schematically shown in Figure 4. As in most of the samples, no significant amount of calcium carbonate could be detected from TGA measurements; carbonation processes were neglected in the calculation of the normalization values for the two different scenarios of this study that constitutes a limitation of the approach. m600^*(1)^ is used instead of m600^*^ to calculate the amount of CH in the sample according to Equation (5):

(6) $${m600}^{*(1)}{=m600}^{*}\;\cdot \;\left(1+\frac{\frac{\mathrm{CH}\_\mathrm{add}}{74\;g/\mathrm{mol}}\;\cdot \;18\;g/\mathrm{mol}}{\frac{\mathrm{CH}\_\mathrm{add}}{74\;g/\mathrm{mol}}\;\cdot \;56\;g/\mathrm{mol}+\mathrm{PC}+\mathrm{MK}}\right)$$

where:

- m600^*(1)^ is the normalization value for scenario 1 in wt.%;
- m600^*^ is the corrected mass at 600 °C taken from TGA data in wt.% (m600 shown in Figure 3, corrected according to Equation (4));
- CH_add is the amount of additionally added CH in the initial binder (t = 0) in wt.%;
- PC is the amount of PC in the initial binder (t = 0) in wt.%;
- MK is the amount of MK in the initial binder (t = 0) in wt.%.

In a next step, the consumed CH by pozzolanic reaction (*Consumed CH*) was calculated according to Equation (7) based on a linear assumption of the produced CH from PC (CH_PC) while considering the replacement ratio of PC by MK. This simplification is critically discussed later, in Section 3.

Subsequently, *Consumed CH* was used to check whether the presumptions of scenario 1 were correct, viz. (CH_add (t = x) = CH_add (t = 0)). If *Consumed CH* was less than CH_PC, the assumption was considered to be correct. If, in contrast, *Consumed CH* was larger than CH_PC, the second scenario was applied and used for further analysis of the TGA data (CH_add (t = x) < CH_add (t = 0)). *CH* in the sample at testing time (t = x) was recalculated by using the adjusted normalization value m600^*(2)^ according to Equation (8). The normalization value in this step was corrected with the stoichiometrically calculated amount of water within the total amount of CH in the sample that was assumed to consist of remaining and not reacted CH_add only, since it is assumed that the CH generated by cement hydration (CH_PC) is completely consumed (m600^*(2)^, Equation (8)).

(7) $$Consumed\;CH=\mathrm{CH}\_\mathrm{PC}+\mathrm{CH}\_\mathrm{add}\;-\;CH=\left(1\;-\;\frac{k}{100}\right)\;\cdot \;\mathrm{CH}\_\mathrm{MK}0+\mathrm{CH}\_\mathrm{add}-\;CH\;$$

where:

- *Consumed CH* is the amount of CH consumed by the pozzolanic reaction of MK in g/100 g binder;
- CH_PC is the amount of CH produced by PC hydration (t = x) in g/100 g binder;
- CH_add is the amount of additionally added CH in the initial binder (t = 0) in g/100 g binder or wt.%, respectively;
- *CH* is the amount of calcium hydroxide in the sample (t = x) in g/100 g binder;
- k is the replacement ratio of PC by MK in wt.%;
- CH_MK0 is the amount of *CH* in the reference sample without MK (t = x) in g/100 g binder (calculated according to Equation (5)).

(8) $${m600}^{*(2)}{=m600}^{*}\;\cdot \;\left(1+\frac{{\mathrm{ML}\_\mathrm{CH}}^{*}}{{m600}^{*}}\right)$$

where:

- m600^*(2)^ is the normalization value for scenario 2 in wt.%;
- m600^*^ is the corrected mass at 600 °C taken from TGA data in wt.% (m600 shown in Figure 3, corrected according to Equation (4));
- ML_CH^*^ is the corrected mass loss between approximately 400 °C and 500 °C determined with the tangential method from TGA data in wt.% (ML_CH shown in Figure 3, corrected according to Equation (4)).

**Figure 5:**
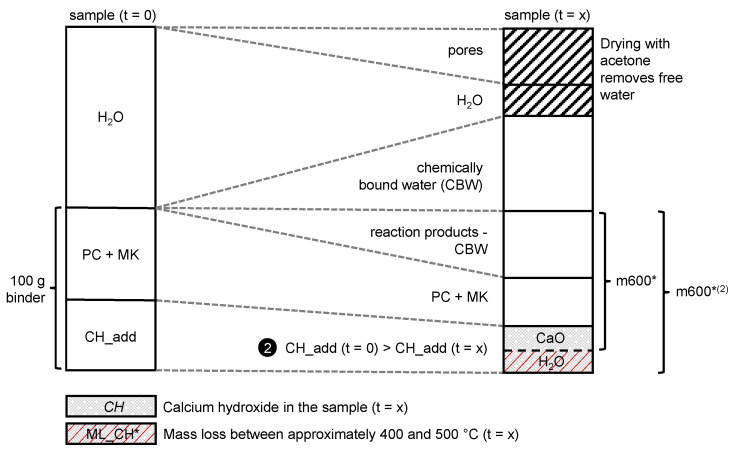
Scheme explaining the calculation of the normalization value m600^*(2)^ for TGA of samples with CH_add according to scenario 2 with the assumption that CH_add is partly consumed by the pozzolanic reaction of MK. The linear connection between t = 0 and t = x is only a simplification and does not play a role for the calculation of the normalization value m600^*(2)^. The dimensions are not realistic; they are chosen for a clear visualization only.

Figure 5 shows schematically how the m600^*(2)^ was calculated. It was used to recalculate *CH* in the sample according to Equation (5) (m600^*^ was replaced by m600^*(2)^). In a next step, the *Consumed CH* was recalculated.

**Figure 6:**
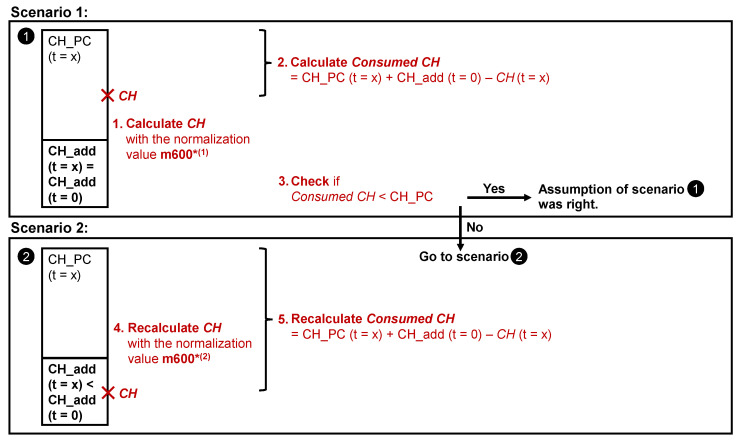
Flow chart explaining the calculation of *Consumed CH* from the TGA data for binder samples with CH_add according to the two different scenarios introduced in this study.

A detailed flow chart of the approach considered in this study for the evaluation of TGA data of the samples with CH_add in the mixture is provided in Figure 6. The upper part of the flow chart shows the subsequent steps to be followed in the first scenario while the lower part shows the consequential steps when the conditions for the first scenario are not fulfilled.

## 3. Results and Discussion

**Figure 7:**
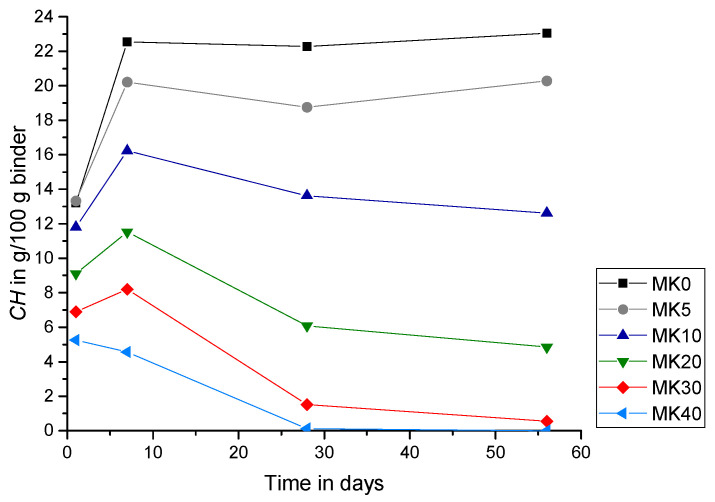
Total amount of *CH* over hydration time (no CH_add).

Figure 7 shows the total amount of CH in the samples (*CH* according to Equation (5)) without the additionally added CH (CH_add) as a function of the hydration time. In the reference sample without metakaolin (MK0), *CH* increases rapidly during the first 7 days of hydration and remains almost constant from 7 until 56 days. In contrast, for the samples containing MK, the amount of CH declines from the age of seven days on, which is due to the pozzolanic reaction. However, it is interesting to mention that, for the sample with 5 wt.% MK replacement (MK5), the pozzolanic reaction after 7 days shows a reduction of CH up to 28 days of hydration, followed by a slight increase in the amount of CH thereafter. This clearly indicates that, for this replacement ratio, the production of CH due to the PC reaction is larger than the consumption of CH due to the pozzolanic metakaolin reaction from 28 days onwards. For the replacement ratios of 10 to 30 wt.% MK (MK10, MK20 and MK30), the total amount of CH in the samples decreases continuously after 7 days of hydration, where for a replacement ratio of 40 wt.% MK (MK40) this decline starts directly after initial hydration, showing a significant shortage of CH right from the start of reaction. The results shown in Figure 7 agree very well with results from literature [21,38,39]. For example, in their study, Wild and Khatib showed that pastes with 15 wt.% cement replacement by MK contain a CH content of around 50% compared to a reference sample without MK at an age of one year [21]. For replacement ratios up to 30 wt.%, an increase in CH in the first week and a reduction after day seven was noted. In the first days, the CH production from cement hydration dominates the pozzolanic reaction of MK as also stated by El-Diadamony et al. [38] for replacement ratios up to 20 wt.% and Fías and Cabrera [39] for ratios of up to 25 wt.% MK.

**Figure 8:**
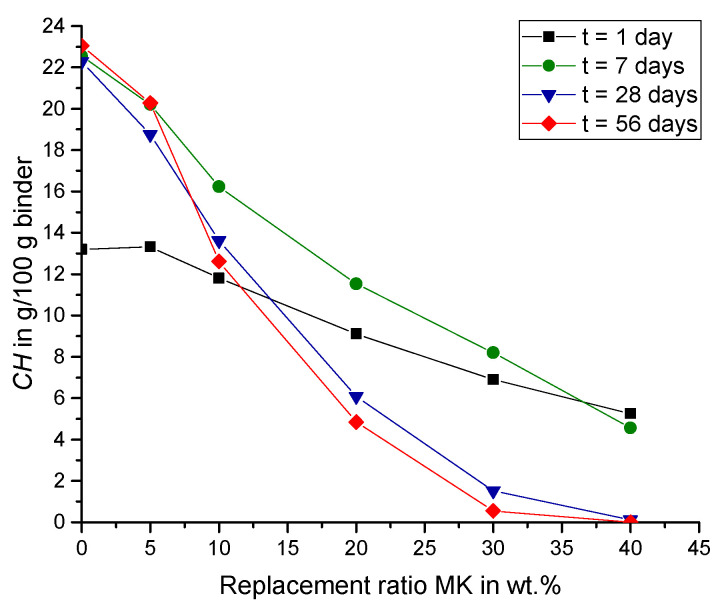
Total amount of *CH* over replacement ratio MK (no CH_add).

Figure 8 shows the same data of *CH* (according to Equation (5)) for 1, 7, 28 and 56 days as a function of the replacement ratio in wt.% MK. The slope is significantly steeper for longer hydration times indicating that the pozzolanic reaction of MK enhances during the first to the seventh day of hydration, and even accelerates up to 28 days. Between 28 and 56 days, however, the reduction of CH due to pozzolanic MK reaction slows down for all samples with the exception of 5 wt.% MK (MK5) as described above.

**Figure 9:**
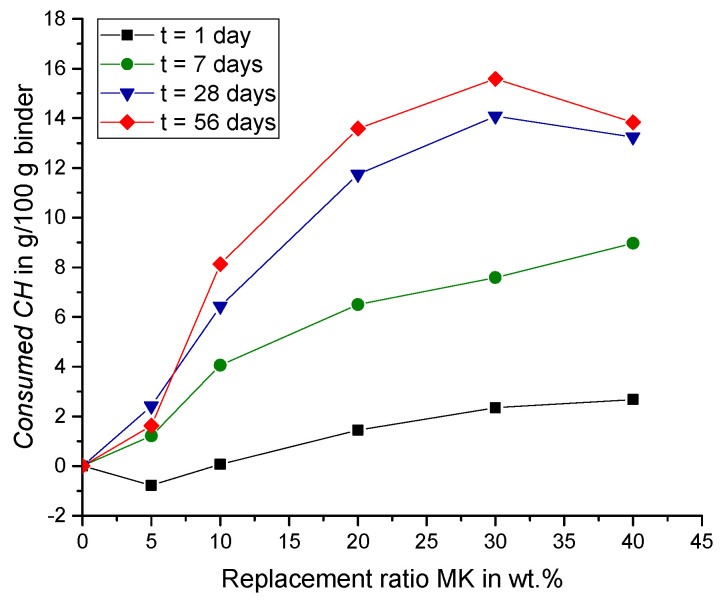
*Consumed CH* over replacement ratio MK (no CH_add).

These findings can also be observed by looking at the *Consumed CH* (according to Equation (7)) shown in Figure 9. The value of *Consumed CH* for a replacement ratio of 5 wt.% MK is slightly negative after one day (−0.78 g/100 g binder), as shown in Figure 9. The negative value indicates that slightly more *CH* was measured in this sample (MK5) compared to the reference sample without MK (MK0). This could be due to a larger amount of fine particles in the system that might cause additional nucleation sites for the hydration products, while promoting the cement hydration [40,41,42]. This nucleation effect is not considered for the calculation of *Consumed CH* (Equation (7)) because of the linear behavior of CH_PC that was assumed with regard to the replacement ratio. Consequently, this may lead to an underestimation of the CH consumption due to pozzolanic reaction of MK. For possible improvements of Equation (7), MK-PC interactions are discussed in more detail in, e.g., Lagier and Curtis [43], who showed that metakaolin may affect cement hydration reactions, depending on the cement composition as well as on the fineness of the metakaolin. However, more research is needed to better understand the reactions and products formed with time. As this information for the different MK dosages is not yet available, a simplified approach proposed in Equation (7) was employed. This equation assumes that the kinetics of the cement generated CH is affected only by the amount of cement in the total binder, i.e., PC replacement ratio, thus neglecting possible MK-PC interaction effects. However, it should be noted that the calorimetric measurements of Lagier and Curtis [43] indicated slight acceleration (nucleation) effects. Moreover, for some cement compositions (also with increased alkali contents), this study also reports additional exothermic reactions, related to an increased reactivity of calcium-aluminate clinker phases and/or metakaolin. However, a more accurate estimation of the amount of CH_PC generated by the (partial) cement reaction will be quite demanding and would require a combination of several measurement techniques (e.g., powder X-ray diffraction, solid state nuclear magnetic resonance, apart from TGA and calorimetry) as well as an advanced mass balance modeling approach.

Figure 9 shows that, after one and seven days of hydration, the *Consumed CH* increases with higher replacement ratios (except from MK5 after one day). The increasing amount of MK leads to a higher pozzolanic reactivity and consequently to an enhanced amount of *Consumed CH*. After seven days, cement hydration and thus the production of CH from PC (CH_PC) slows down, while the pozzolanic reaction of MK endures. After 28 and 56 days, the *Consumed CH* is largest for a replacement ratio of 30 wt.% MK. For a replacement ratio of 40 wt.%, no *CH* could be detected by thermogravimetric analysis at all, after 28 days of hydration (Figure 7 and Figure 8). Consequently, the pozzolanic reaction in these samples are restrained by the limited amount of CH_PC, so that the *Consumed CH* decreases for samples with more than 30 wt.% MK. These findings are in line with observations from the literature. For example, Khater [23] attributes the decreasing bulk density of mortar samples with a replacement ratio of 30 wt.% MK to the pozzolanic reaction [23]. Results from Aramburo et al. on mortar samples prepared with high replacement ratios of PC, exchanging it with a mixed calcined clay (MK content ~45 wt.%), showed an increased compressive strength for a replacement ratio of 40 wt.% calcined clay (replacement ratio of PC by MK ~23 wt.%), but a decreased compressive strength for a replacement ratio of 50 wt.% (replacement ratio of PC by MK ~31 wt.%) and higher [28]. With incorporating CH_add in the mix, the compressive strength could increase for these samples as well, while assuming that the reduction of strength for high replacement ratios is due to a lack of CH that affects the pozzolanic reaction of the reactive calcined clay [28]. Moreover, Sha and Pereira noted that samples with replacement ratios of 30 wt.% MK in cement pastes showed a diminishing peak of CH in differential scanning calorimetry thermograms with age [44].

**Figure 10:**
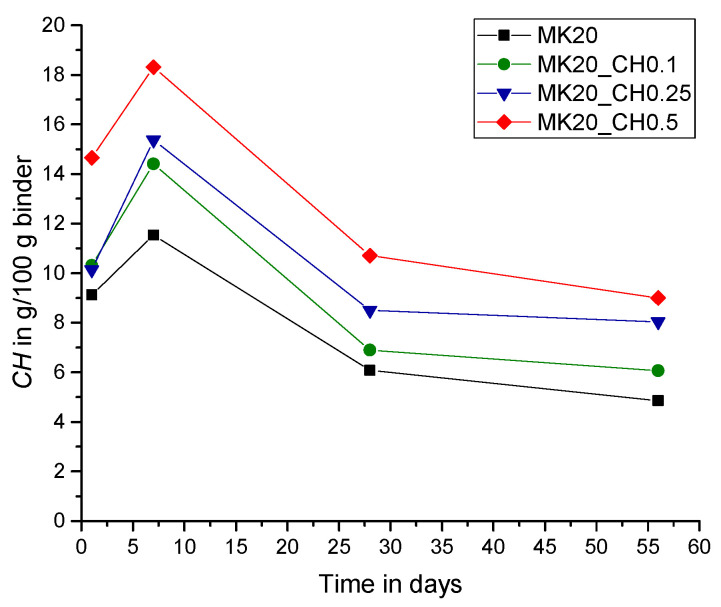
Total amount of CH over hydration time (CH_add/MK = 0.1).

**Figure 11:**
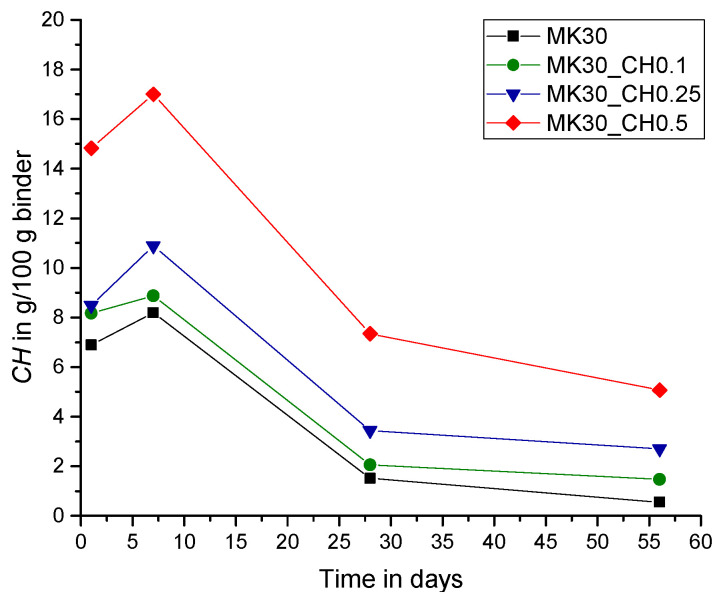
Total amount of CH over hydration time (CH_add/MK = 0.25).

**Figure 12:**
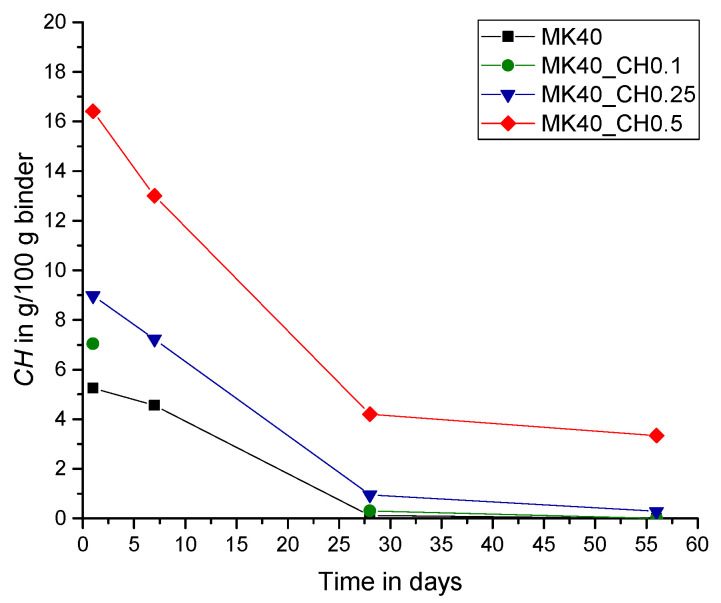
Total amount of CH over hydration time (CH_add/MK = 0.5).

**Figure 13:**
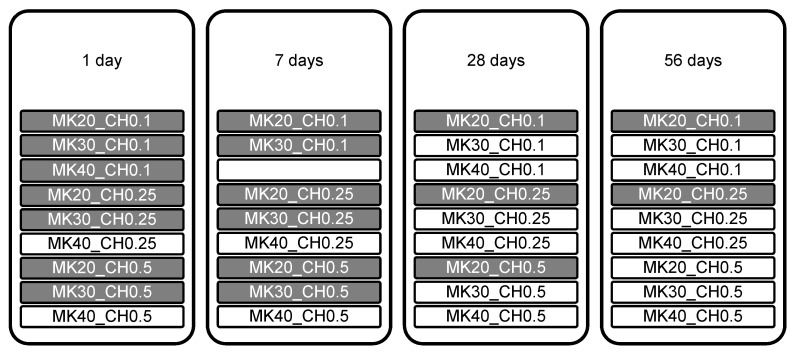
Cement paste samples of the experimental program. Samples of scenario 1, where CH_add was not needed for pozzolanic reaction of MK and it consequently remained unreacted, are marked with a grey background. Samples of scenario 2, with complete consumption of CH_PC by pozzolanic reaction of MK, are marked with a white background.

In the present study, the lack of CH in the paste systems was compensated by additional amounts of powder calcium hydroxide with CH_add/MK ratios of 0.1, 0.25 and 0.5. The results in Figure 10, Figure 11 and Figure 12 show the measured amount of *CH* (according to Equation (5)) in the samples with the additionally added CH (CH_add) with hydration time. For the sample MK40_CH0.1 an error appeared in the measurement after seven days of hydration and it is thus excluded from Figures 12, 13 and 15. Similar to the results above, the total amount of CH decreases with increasing replacement ratios of PC by MK. As observed, the incorporation of CH_add leads to higher values of *CH* in the samples. Similar to the results in Figure 7 without CH_add, Figure 10, Figure 11 and Figure 12 show that a replacement ratio of 40 wt.% MK results in a reduction of CH right from the first day. With replacement ratios of 20 and 30 wt.%, an increase in CH is noted until the seventh day, followed by a rapid decrease with hydration time.

Following the mass balance approach for the calculation of *Consumed CH* described in Section 2.4, Figure 13 provides an overview of the samples and their assignment to scenario 1 and 2. Samples that were analyzed following scenario 1 assuming no consumption of CH_add by the pozzolanic reaction of MK are marked in grey (Figure 13). In these cases, only CH produced by PC (CH_PC) reacted (partially) with MK and CH_add remained unreacted. In contrast, samples assigned to scenario 2 are shown in white boxes (Figure 13). In these samples, CH_PC was fully consumed by the pozzolanic MK reaction, and/or CH_add partially reacted. Figure 13 shows that up to a hydration time of seven days, CH_add reacted only when PC was replaced by 40 wt.% MK. From 28 days onwards, the results showed that the amount of CH_PC was not enough for samples with replacement ratios of 30 and 40 wt.% MK. The results show a complete consumption of CH_PC by the pozzolanic MK reaction for replacement ratios of at least 30 wt.% and with extra CH added with CH_add/MK ratios of 0.1 and higher, from 28 days on. This mixture (MK30_CH0.1) contains an amount of 67.96 wt.% PC (Table 2) representing a threshold value for the complete consumption of CH_PC by MK reaction after 28 days. After 56 days, a threshold value of 72.73 wt.% PC (Table 2) is given by the sample MK20_CH0.5, according to Figure 13.

**Figure 14:**
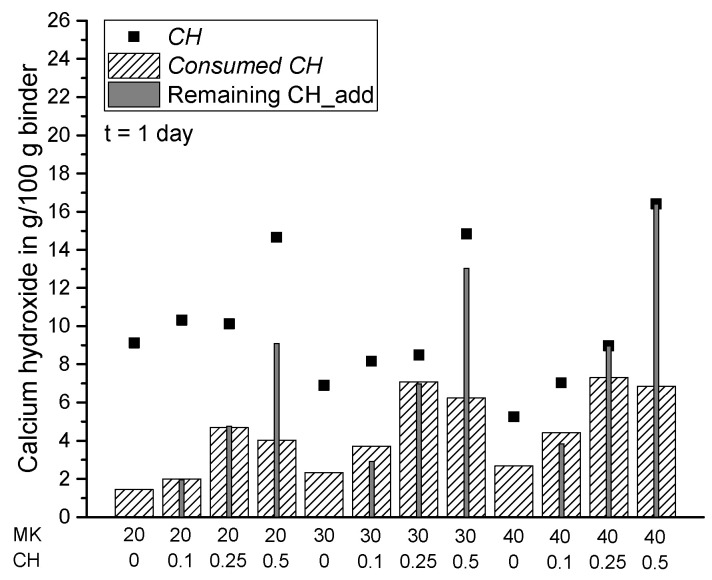
Summary of *CH*, *Consumed CH* and remaining CH_add for each sample after one day of hydration.

**Figure 15:**
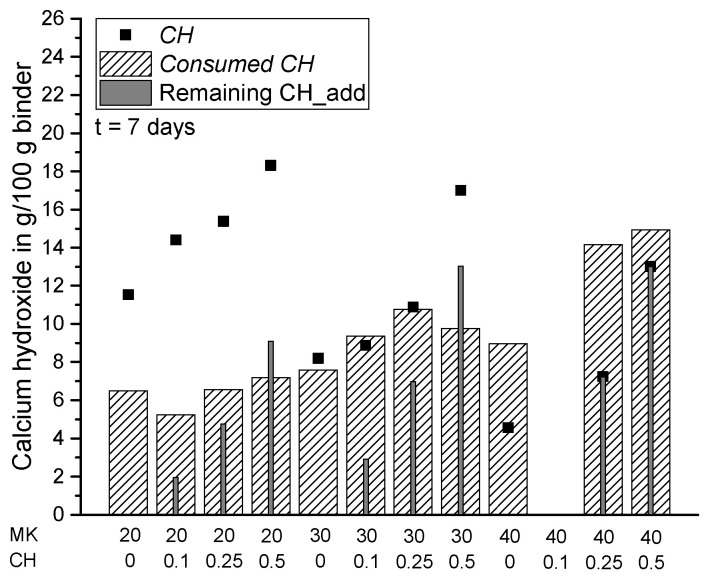
Summary of *CH*, *Consumed CH* and remaining CH_add for each sample with the exception of MK40_CH0.1 after 7 days of hydration.

**Figure 16:**
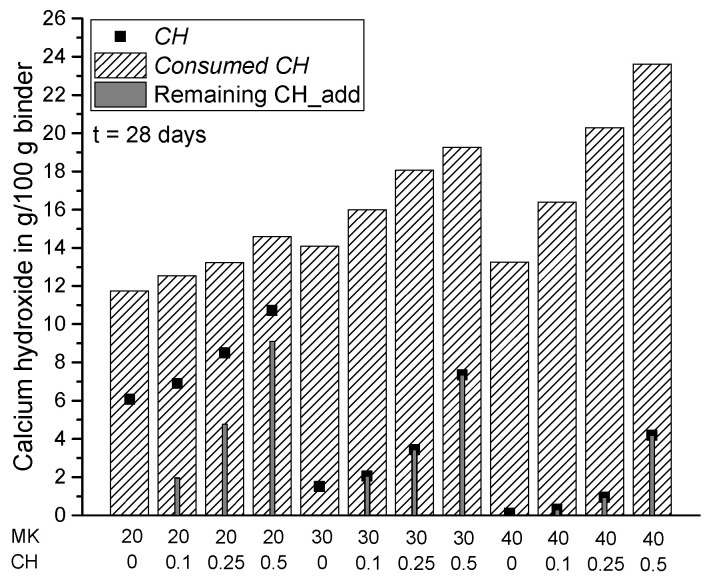
Summary of *CH*, *Consumed CH* and remaining CH_add for each sample after 28 days of hydration.

**Figure 17:**
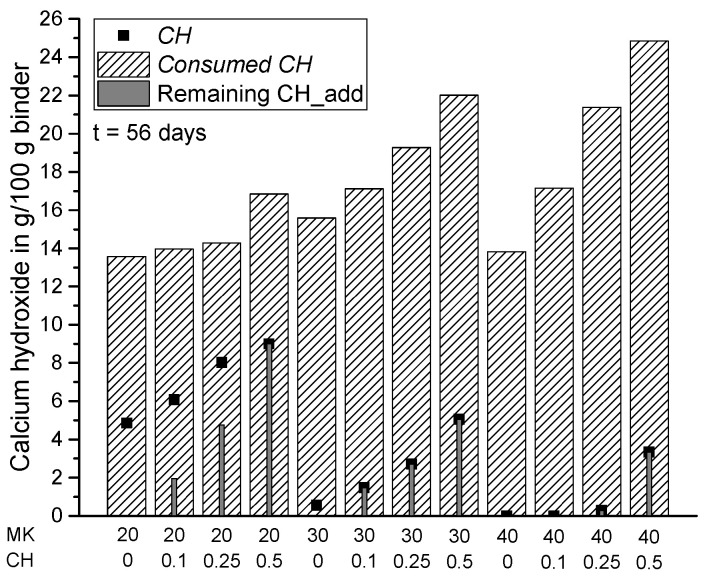
Summary of *CH*, *Consumed CH* and remaining CH_add for each sample after 56 days of hydration.

Figure 14, Figure 15, Figure 16 and Figure 17 summarize the total amount of *CH* measured in the samples (according to Equation (5)), the consumption of CH by pozzolanic reaction of MK (*Consumed CH* according to Equation (7)) and the amount of additionally added but not reacted CH in the samples (remaining CH_add) at the various investigated hydration times. For the samples from scenario 1, the remaining CH_add is equal to the initially added CH (CH_add). For the samples assessed according to scenario 2, the remaining CH_add can be calculated as follows: CH_add (t = 0) + CH_PC (t = x) − *Consumed CH* (t = x). In comparison with Equation (7), it becomes clear that *CH* has the same value as the remaining CH_add in scenario 2 resulting from the assumption of a complete consumption of CH_PC, so that the measured *CH* contains additionally added, but not reacted CH (remaining CH_add) only. For this reason, these two parameters have the same value for all samples corresponding to scenario 2 (Figure 14, Figure 15, Figure 16 and Figure 17). The results confirm that the total amount of CH in the matrix can be increased by adding extra CH in the mixture. Based on the results of *Consumed CH*, this study also shows that the pozzolanic reaction of MK can be enhanced by incorporating CH_add for high replacement ratios ranging from 20 to 40 wt.% MK.

**Figure 18:**
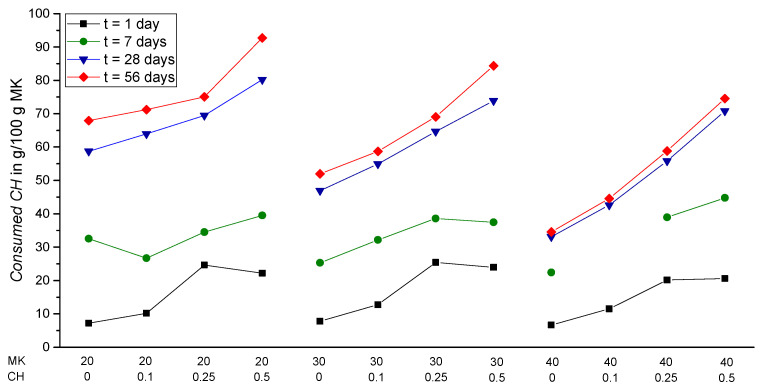
*Consumed CH* in g/100 g MK for each sample with the exception of MK40_CH0.1 (t = 7 days) and the different hydration times.

This finding can even be strengthened more when comparing the *Consumed CH* to the initial amount of MK in the binder, as shown in Figure 18 (results in units of g/100 g MK), for all specimens of four different ages. For higher amounts of CH_add, the *Consumed CH* by pozzolanic MK reaction increases especially after 28 and 56 days. At a first glance, this turned out to be in contrast to a study published by Briki et al. that shows the degree of reaction of a cement paste system with calcined clay (~48 wt.% metakaolin) and the additional incorporation of calcium hydroxide (CH_add/MK ~0.36). In comparison with the samples without CH_add, their results showed an increase in the reaction degree up to the seventh day of hydration, but had a similar reaction degree at later ages [32]. Following these results, Briki et al. concluded that a lack of CH cannot be the reason for slowing down the metakaolin reaction in LC³-systems at later ages [32]. Regarding the influence of CH_add on the MK reaction, the main differences between the results published by Briki et al. and the results reported in this study can be explained by the difference in binder compositions used in both experiments. Namely, Briki et al. used a LC³-system containing clinker (47.8 g/100 g solid), calcined clay (28.6 g/100 g solid), limestone (19.0 g/100 g solid), calcium hydroxide (5.0 g/100 g solid) and anhydrite (4.5 g/100 g solid). Therefore, to make these results comparable with the results published in this paper, only the parts of cement and metakaolin should be considered. As their calcined clay contained approx. 48 wt.% metakaolin, the replacement ratio of cement against MK turned out to be around 22.3 wt.% [(0.48 · 28.6 g)/((0.48 · 28.6 g) + 47.8 g)]. When comparing this replacement ratio (viz. 22.3 wt.%) with the replacement ratio of 20 wt.% of this study, it turns out that for this replacement ratio there is still sufficient CH_PC available for the pozzolanic reaction of MK and CH_add is not consumed. Moreover, the relatively “low” replacement ratio of around 22.3 wt.% along with further synergetic effects caused by limestone in the LC³-system of Briki et al. [32] could possibly be the reason for the different findings on the metakaolin reactivity in cement pastes with additionally added CH.

Figure 18 also shows that the amount of *Consumed CH* relative to the amount of MK decreases for increasing replacement ratios after 28 and 56 days. The values of *Consumed CH* in g/100 g MK are lower at a replacement ratio of 30 wt.% from 28 to 56 days than at a replacement ratio of 20 wt.% (Figure 18). Even though some remaining CH_add was still found in these samples after 28 days (Figure 16), the amount of *Consumed CH* by MK is less, compared to a replacement ratio of 20 wt.%. This effect could be due to the relatively lower amount of PC that results in a lower availability of CH in the binder. Moreover, in the samples with 20 wt.% MK, the calcium hydroxide demand of the MK reaction up to 28 days of hydration is still sufficiently supplied by CH_PC and CH_add is not consumed at all (Figure 13). In contrast, CH_PC is completely consumed in the samples with 30 wt.% MK after 28 days and CH_add takes part in the pozzolanic reaction of MK (Figure 13). This finding could indicate that the pozzolanic reaction of MK with CH_add is somehow weaker compared to the reaction with CH_PC. The low values of *Consumed CH* in g/100 g MK for the replacement ratio of 40 wt.% after 28 and 56 days in Figure 18 can be explained by a lack of CH in the samples as already shown in Figure 16 and Figure 17, respectively. After 56 days and a replacement ratio of 40 wt.%, only the samples with additional CH and CH_add/MK ratios of 0.25 and 0.5 have a little remaining content of *CH* of 0.3 g/100 g binder and 3.3 g/100 g binder, respectively (Figure 17). The lack of CH also explains the small difference between the measured values of *Consumed CH* for samples with a replacement ratio of 40 wt.% MK from 28 to 56 days (Figure 18). Regarding the samples with a replacement ratio of 20 wt.% MK, the smaller value of *Consumed CH* after 7 days with a CH_add/MK ratio of 0.1 (MK20_CH0.1) compared to the sample with no CH_add (MK20) cannot be explained with the data from this study.

The results show that the pozzolanic reaction of MK is limited by the availability of calcium hydroxide generated by cement hydration (CH_PC) for replacement ratios of 30 wt.% and higher. The addition of extra CH in the initial mixture could possibly enable a better strength performance for increased replacement ratios of PC by MK due to an enhanced pozzolanic reaction of MK. For replacement ratios of 30 and 40 wt.% and the addition of extra CH with a CH_add/MK ratio of 0.25 and 0.5, respectively, the same reactivity of MK as compared to a replacement ratio of 20 wt.% without CH_add can be evaluated based on the *Consumed CH* in g/100 g MK (Figure 18). Regarding the amount of CH in the cement-based binder, adding extra CH to a mixture could be an interesting approach, especially when a minimum amount of CH should remain in the matrix to guarantee sufficient (buffering) alkalinity, e.g., for the passivation of steel in reinforced concrete. Assuming a minimum amount of CH of, e.g., 2.6 g/100 g binder in the matrix [45], Figure 17 shows that a replacement ratio of 20 wt.% can be realized without CH_add up to 56 days of hydration. However, for a replacement ratio of 30 wt.% a minimum of CH_add/MK ratio of 0.25 (*CH* = 2.7 g/100 g binder) and for a 40 wt.% ratio of at least 0.5 (*CH* = 3.2 g/100 g binder) is required.

## 4. Conclusions

In this study, increased replacement ratios of Portland cement (PC) by metakaolin (MK) were studied, with emphasis on the effect of adding additional calcium hydroxide (CH_add). A mass balance approach was employed for normalization and analysis of thermogravimetric data, and the results are expressed relative to the total amount of binder, which also contains CH_add. The main outcomes of this study are summarized below:

- For replacement ratios of 20 and 30 wt.% MK, an increase in calcium hydroxide (CH) is observed until the seventh day, followed by a decrease during further hydration time. For the samples with a replacement ratio of 40 wt.% MK, the reduction of CH already starts at the first day.
- For samples without CH_add, CH consumption by pozzolanic reaction of MK after 28 and 56 days is the highest with a replacement ratio of 30 wt.% MK.
- For samples without CH_add, replacement ratios of 30 wt.% MK or higher result in a complete consumption of CH after 28 days at the latest so that the pozzolanic reaction of MK is limited by the amount of CH supplied by cement hydration in these samples.
- CH_add leads to a stronger pozzolanic reaction of MK based on the calculated CH consumption.

## Figures and Tables

**Table 1:** Chemical compositions of the used metakaolin (MK) and Portland cement (PC) in wt.% (evaluated on pressed tablets by X-ray fluorescence analysis).

	SiO_2_	Al_2_O_3_	Fe_2_O_3_	CaO	MgO	Na_2_O	K_2_O	TiO_2_	Other
MK	52.95	42.18	2.38	0.05	0.07	0.00	0.31	1.77	0.29
PC	19.40	3.30	5.42	68.58	0.69	0.27	0.47	0.24	1.63

**Table 2:** Abbreviation and compositions of paste samples.

Abbreviation	Replacement Ratio in wt.%	CH_add/MK	MK in wt.%	PC in wt.%	CH_add in wt.%
MK0	0	-	0.00	100.00	0.00
MK5	5	-	5.00	95.00	0.00
MK10	10	-	10.00	90.00	0.00
MK20	20	-	20.00	80.00	0.00
MK30	30	-	30.00	70.00	0.00
MK40	40	-	40.00	60.00	0.00
MK20_CH0.1	20	0.10	19.61	78.43	1.96
MK20_CH0.25	20	0.25	19.05	76.19	4.76
MK20_CH0.5	20	0.50	18.18	72.73	9.09
MK30_CH0.1	30	0.10	29.13	67.96	2.91
MK30_CH0.25	30	0.25	27.91	65.12	6.98
MK30_CH0.5	30	0.50	26.09	60.87	13.04
MK40_CH0.1	40	0.10	38.46	57.69	3.85
MK40_CH0.25	40	0.25	36.36	54.55	9.09
MK40_CH0.5	40	0.50	33.33	50.00	16.67

## Data Availability

The data presented in this study are available on request from the corresponding authors.
